# A systematic review of health state utility values for thyroid cancer

**DOI:** 10.1007/s11136-020-02676-2

**Published:** 2020-10-24

**Authors:** Rachel Houten, Nigel Fleeman, Eleanor Kotas, Angela Boland, Tosin Lambe, Rui Duarte

**Affiliations:** 1 Liverpool Reviews and Implementation Group, University of Liverpool, 2.06 Whelan Building, The Quadrangle, Brownlow Hill, Liverpool, L69 3GB UK; 2 York Health Economics Consortium, University of York, York, UK

**Keywords:** Health-related quality of life, Systematic review, Thyroid cancer, Utility values

## Abstract

**Purpose:**

Health state utility values are commonly used to inform economic evaluations and determine the cost-effectiveness of an intervention. The aim of this systematic review is to summarise the utility values available to represent the health-related quality of life (HRQoL) of patients with thyroid cancer.

**Methods:**

Eight electronic databases were searched from January 1999 to April 2019 for studies which included assessment of HRQoL for patients with thyroid cancer. Utility estimates derived from multiple sources (EuroQol questionnaire 5-dimension (EQ-5D), time trade-off [TTO] and standard gamble [SG] methods) were extracted. In addition, utility estimates were generated by mapping from SF-36 and EORTC QLQ-30 to the EQ-5D-3L UK value set using published mapping algorithms.

**Results:**

Searches identified 33 eligible studies. Twenty-six studies reported HRQoL for patients with differentiated thyroid cancer and seven studies for patients with general thyroid cancer. We identified studies which used different methods and tools to quantify the HRQoL in patients with thyroid cancer, such as the EQ-5D-3L, SF-36, EORTC QLQ-30 and SG and TTO techniques to estimate utility values. Utility estimates range from 0.205 (patients with low-risk differentiated thyroid cancer) to utility values approximate to the average UK population (following successful thyroidectomy surgery and radioiodine treatment). Utility estimates for different health states, across thyroid cancer sub-types and interventions are presented.

**Conclusion:**

A catalogue of utility values is provided for use when carrying out economic modelling of thyroid cancer; by including mapped values, this approach broadens the scope of health states that can be considered within cost-effectiveness modelling.

**Electronic supplementary material:**

The online version of this article (10.1007/s11136-020-02676-2) contains supplementary material, which is available to authorised users.

## Introduction

Thyroid cancer is the most common type of endocrine-related cancer [1]. There has been a continuous rise in the incidence of thyroid cancer over the last few decades in various countries worldwide [2]. In 2015, there were 3,528 new cases [3] of thyroid cancer in the UK, with incidence rates projected to rise by 74% between 2014 and 2035; this is equivalent to 11 cases per 100,000 people [4].

Thyroid cancers are divided into four main sub-types. Papillary (PTC) and follicular thyroid cancers (FTC) are commonly grouped as differentiated thyroid cancer (DTC) and account for approximately 94% [5] of thyroid cancers. Anaplastic thyroid cancer (ATC) is the most severe type of thyroid cancer and accounts for about 2% of thyroid cancers. Medullary thyroid cancer (MTC) accounts for the remaining patients diagnosed with thyroid cancer [6].

Treatment depends on the sub-type of thyroid cancer; however, initial therapy often involves a partial or total thyroidectomy. Radioactive iodine therapy and radiotherapy may also be offered to reduce the risks of recurrence [6]. Targeted cancer therapies and chemotherapies may be offered in cases of advanced cancer, or relapsing cancer that is symptomatic [7]. Symptoms associated with relapsing thyroid cancer include neck swelling, neck pain, trouble breathing or swallowing, hoarseness or other voice changes [8]. Many of these treatments are likely to have a substantial impact on health-related quality of life (HRQoL). Many people diagnosed with thyroid cancer are young and need lifetime monitoring and surveillance; there are therefore many negative impacts on HRQoL including anxiety associated with disease recurrence, disruptions to social life and changes to physical appearance [9].

Several of the health technology assessment (HTA) organisations in Europe require an economic evaluation to be submitted as part of the evidence base used to appraise health technologies, with effectiveness expressed in quality adjusted life years (QALYs). QALYs can be used to represent both the quality and quantity of life in each health state described in an economic evaluation, where a health state represents aspects of the condition treated by the health technology e.g. disease-free, progressed disease or post-surgery.

The HRQoL element of the QALY is measured in terms of utility values. HTA organisations, such as the National Institute for Health and Care Excellence (NICE) and the Scottish Medicines Consortium (SMC) stipulate that utility should be measured using the EuroQol questionnaire 5-dimension (EQ-5D) [10]. The EQ-5D is a generic preference-based HRQoL questionnaire. It is completed by the patient population of interest and the responses to the questionnaire are then weighted according to the preferences of the general population. Non-preference-based HRQoL questionnaires reflect the health of the respondents without any adjustments being made for the preferences of health states as valued by the general public or patients. Utility values are derived from responses to the EQ-5D by applying weights that reflect population preferences for a particular health state. Utility values range from 1 (equivalent to perfect health) to 0 (equivalent to death), with negative values reflecting health states worse than death. Utility values are used to estimate QALYs by reflecting the time spent in a given health state (e.g. the QALY for an individual with 10 years of life at a utility of 0.8 would be equivalent to 8 QALYs).

NICE and the SMC prefer to consider utility estimates derived from the EQ-5D as it is a generic questionnaire and the estimates generated can be compared across diseases. The EQ-5D can be used to generate preference-based weighted utility estimates from the perspective of the general public; i.e. from the people who pay for the NHS, as the NHS is funded by UK taxpayers.

There are two versions of the EQ-5D questionnaire, one with three levels (EQ-5D-3L) and one with five levels (EQ-5D-5L). The EQ-5D questionnaire comprises five dimensions (mobility, self-care, usual activities, pain/discomfort and depression/anxiety). The EQ-5D-3L has three response levels for each of the dimensions: no problems, some problems and extreme problems; the EQ-5D-5L has five response levels: no problems, slight problems, moderate problems, severe problems, unable to/extreme problems. NICE and the SMC prefer utility estimates derived using the EQ-5D-3L UK value set, therefore if the EQ-5D-5L is used in a study to collect HRQoL data then estimating utilities by converting the responses to the EQ-5D-3L UK value set is recommended.

In the absence of directly measured EQ-5D-3L values, economists can use utility estimates that have been generated by ‘mapping’ from other generic HRQoL questionnaires to EQ-5D-3L values. This method involves using a dataset containing both EQ-5D-3L responses and the alternative HRQoL questionnaire responses to generate a mathematical relationship between the responses to the two questionnaires. The regression equation generated can be used to convert the responses to the alternative HRQoL questionnaire into EQ-5D-3L utility estimates. Mapping algorithms are designed to be used with individual patient-level data to incorporate some of the uncertainty associated with estimating HRQoL within a cohort. However, mapping using mean values can offer an alternative in the absence of patient-level data [11]. Values from alternative methods of eliciting utility estimates, for example, using time trade-off (TTO) or standard gamble (SG) approaches, may also be used if EQ-5D-3L utility estimates are unavailable. TTO and SG are preference-based and the preferences (utilities) can be derived from patients for their own health or for scenarios, or from members of the general public for scenarios.

To date, EQ-5D-3L utility values sourced directly from patients with thyroid cancer have been largely absent in economic evaluations of treatments for this population. Due to the increasing incidence of thyroid cancer, there has been a rise in the demand for targeted cancer therapies which has led to a growing number of drugs receiving market authorisation for a thyroid cancer indication [12]. The International Society for Pharmacoeconomics and Outcomes Research (ISPOR) guidelines for good practice when selecting HRQoL values for use in economic modelling [11] highlight that systematic reviews are seldom conducted to inform utility values. This systematic review aims to summarise the utility values available to represent the HRQoL of patients with thyroid cancer with the addition of utility estimates based on mapping non-preference-based HRQoL questionnaires. The objectives of this systematic review are (a) to provide a catalogue of utility values that could be used to inform economic evaluations of thyroid cancer treatments and (b) to identify any potential health states for which published utility estimates are unavailable. Comparisons of utility values across health states are made easy with a catalogue and we include some of our observations as part of this review.

## Methods

This systematic review follows the Centre for Reviews and Dissemination (CRD) guidance on conducting systematic reviews in healthcare [13].

### Search strategy

The search strategy included thyroid cancer terminology and a HRQoL search filter [14] (Supplementary Appendix A) and was adapted according to the specifications of each of the databases utilised. Eight electronic databases were searched: MEDLINE (including MEDLINE In-Process and Other Non-Indexed Citations), EMBASE, Cochrane Database of Systematic Reviews (CDSR), Cochrane Database of Abstracts of Reviews of Effects (DARE), Cochrane HTA database, NHS Economic Evaluation Database (EED), Cost-effectiveness Analysis Registry, Patient-reported outcome and quality-of-life instruments database, as well as the EQ-5D-3L website. The date span of the searches was 1st January 1999 to 6th April 2019. The reference lists of relevant publications and websites were hand-searched to identify additional studies. The results of the searches were uploaded to an Endnote X7.4 library and de-duplicated.

The websites of NICE and the SMC were searched for guidance relating to treating people with thyroid cancer. The purpose of these searches was to identify any utility values included in UK policy documents that had not yet been published in peer-reviewed journals.

### Study selection and inclusion criteria

All publications that described patients with thyroid cancer, of any sub-type, were included in the review if utility estimates were reported or if they could be calculated from the reported mean values. The NICE Reference Case [15] stipulates the use of the EQ-5D tool (3 or 5 level versions) as the preferred HRQoL questionnaire, using the EQ-5D-3L value set to weight the questionnaire responses. Therefore, we identified publications that either directly reported EQ-5D utility estimates or reported HRQoL values measured with a questionnaire that could be used to indirectly estimate utility values using published mapping algorithms (i.e. SF-36 and EORTC QLQ-30). A list of the domains and response level on the EQ-5D-3L, EQ-5D-5L, EORTC QLQ-C30 and SF-36 is presented in Supplementary Appendix B. Studies which elicited utility values using TTO or SG methods were also included.

The EQ-5D has a number of valuation sets from different countries as the preferences for the health states assessed by the EQ-5D vary according to nationality. The studies were not restricted by country of origin.

In order for a study that reported HRQoL values measured by SF-36 or EORTC QLQ-30 questionnaires to be included, either (a) the authors must have mapped the values using algorithms that converted the estimates into EQ-5D-3L utility estimates using the UK value set or (b) it must have been possible to do this ourselves from the data presented.

Study selection was performed by a single reviewer (RH). An inclusive strategy was adopted at the title and abstract screening stage meaning that publications that met the inclusion criteria (Table 1), as well as publications for which there was some uncertainty about the inclusion decision based on their title or abstract alone, were retrieved and judged based on their full text. Two reviewers independently assessed eligibility of the full-text papers retrieved. Any disagreements were resolved through discussion, and, if necessary, in consultation with a third reviewer.

**Table 1:** Inclusion criteria

Criteria	Inclusion
Population	Adults with thyroid cancer
Outcomes	Utility values obtained from EQ-5D-3L questionnaires, HRQoL questionnaires which can be converted to EQ-5D-3L utility estimates (HUI, SF-36, EORTC QLQ-C30), or from TTO and SG studies
Study design	Studies designed to specifically collect HRQoL data, or as part of an RCT or prospective observational study
Date	Clinical and economic searches from January 1999 to April 2019
Language	English language only

*EORTC QLQ-C30* European organisation for the research and treatment of cancer quality-of-life questionnaire for cancer, *EQ-5D-3L* EuroQol questionnaire 5-dimension 3 level version, *HRQoL* health-related quality of life, *HUI* health utilities index, *SF-36* short-form 36-item version, *RCT* randomised controlled trial, *SG* standard gamble, *TTO* time trade-off

### Data extraction and synthesis

Descriptive and methodological characteristics of the studies were extracted. For example, data fields included: sample characteristic, sub-type of thyroid cancer, country of origin, interventions assessed, study design, some details of the patient groups for which HRQoL data were collected, sample size, method of HRQoL data collection (e.g. generic preference-based questionnaire, TTO), and the health states for which utility values were available or could be calculated.

The results were ‘mapped’ onto the utility scale using published mapping algorithms [16, 17] to convert the scores using alternative HRQoL measurement tools to the EQ-5D-3L UK index. Where HRQoL domains included in the published mapping algorithms were not reported in the published studies they were excluded from the review as the utility mapping could not be completed. Data extraction and the mapping of published values from HRQoL questionnaires to the EQ-5D-3L UK value set were performed by two reviewers (TL and RH) independently and the results were then cross-checked. Discrepancies in the results were then discussed by both reviewers and resolved.

A hierarchy of data extraction was developed for those studies reporting multiple HRQoL estimates. Only one set of utility values was extracted from each study. Directly reported utility values from the EQ-5D took precedence, followed by questionnaire data that could be mapped to the EQ-5D-3L using published algorithms [16, 17] and finally data obtained through the use of validated methodologies such as SG or TTO. If, for example, a study reported EQ-5D and SF-36 values, only the EQ-5D values were extracted.

Data from studies that reported only HRQoL changes from a baseline value were also extracted or mapped to the EQ-5D-3L to give a more complete picture of the evidence available. Change from baseline estimates of utility can be useful in economic modelling to populate a model in the absence of health state utility estimates and to validate model outcomes; however, the baseline characteristics of the study populations must be considered to ensure the change estimate is appropriate. Utility values reported for reference purposes only, such as the HRQoL of the general population, were not extracted from the included studies.

A narrative synthesis was used to summarise the information extracted from the included studies; utility values were grouped according to thyroid cancer sub-type and then by the health states that were most similar to each other. More formal methods of synthesis such as meta-analysis could not be used as multiple estimates for sufficiently similar populations were not available.

## Results

**Fig. 1:**
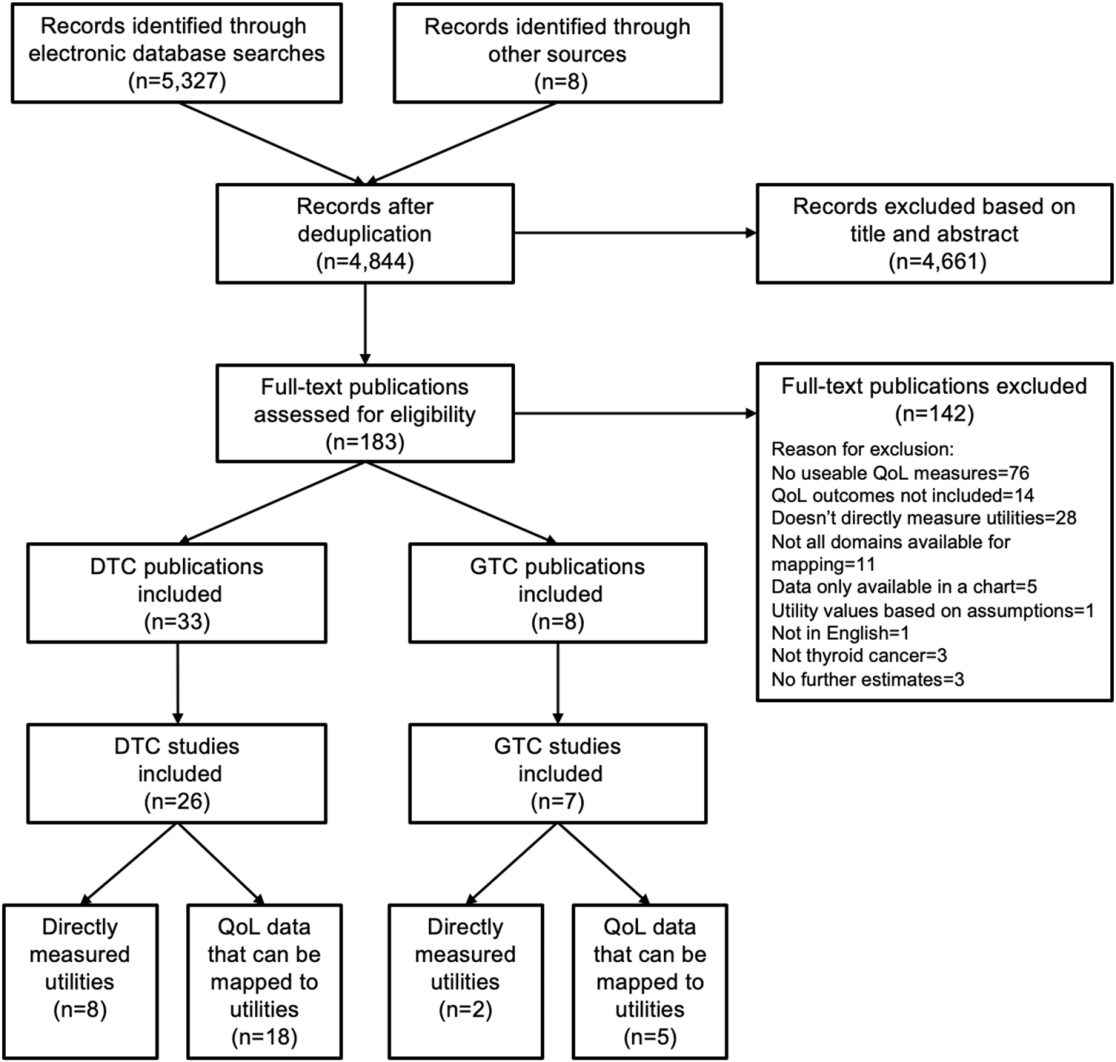
PRISMA flow diagram. *DTC* Differentiated thyroid cancer, *GTC* general thyroid cancer, *MTC* medullary thyroid cancer, *QoL* quality of life

The searches of the electronic databases identified 5327 citations (Fig. 1). Following the screening of titles and abstracts, 183 full-text publications were retrieved for a detailed assessment of eligibility. Consideration of the full-text papers led to the exclusion of 142 publications. The remaining 41 publications (33 studies) [1, 9, 18–57] formed the final set of evidence included in this systematic review.

The evidence was reported according to the sub-type of thyroid cancer studied (for example, PTC and FTC were grouped as DTC). There were no publications that described patients specifically with ATC or MTC; however, the general thyroid cancer (GTC) group included patients with several sub-types of thyroid cancer or included patients for whom the sub-type of thyroid cancer was not reported. A summary of the characteristics of all of the included publications is presented in Table 2. The sample characteristic column provides details of the population in which HRQoL is measured.

**Table 2:** Characteristics of publications that were included in the HRQoL evidence review

Sample characteristic	Publication	Population/country	Intervention	Study design	Sample size	Type of HRQoL assessment tool	Specific HRQoL assessment tool used	Reported health states	Response rate	Missing data
DTC										
HRQoL assessed in a whole population (no specific intervention)										
UK general population asked to evaluate thyroid cancer health states	Fordham [46] and Kerr (abstract) [47]	Population: RR-DTC Country: UK	None	Vignette health states evaluated through asking a sample of the general public	*N* = 100	TTO interviews	Vignette health states evaluated via interview	Observed (unadjusted) utility values: stable/no response, response to therapy, progressive disease, diarrhoea, fatigue, hand and foot syndrome, alopecia	NR	NR
Patients with DTC admitted to hospital	Haraj [55]	Population: DTC admitted to hospital Country: Morocco	None	Observational cross-sectional	*N* = 124	Patient-reported survey	SF-36	Average HRQoL across DTC population	NR	NR
Patients with long-standing DTC	Hedman (abstract) [24] Hedman [25] (Full publication of Hedman abstract) Hedman [17]	Population: DTC (14–17 years post-diagnosis) Country: Sweden	None	Prospective	*N* = 279	Patient-reported survey	SF-36	Average HRQoL across DTC population, sub-groups according to number of comorbidities, self-reported recurrence and general attitude to life; major, moderate or no thyroid-related symptoms; patients with and without thyroid symptoms	79%	SF-36 scoring software used to handle missing values
Patients with long-standing DTC	Hedman [54]	Population: DTC (diagnosed between 2012–2017) Country: Sweden	None	Prospective	*N* = 487	Patient-reported survey	SF-36	Average across all patients at diagnosis and a year after diagnosis, sub-groups according to TSH suppression, attitude to life and fear of recurrence, presence of recurrence	72% (67% at one-year follow-up)	SF-36 scoring software used to handle missing values
Attendees at patient–doctor thyroid cancer forum	McIntyre [51]	Population: DTC with a diagnosis at least 6 months prior to assessment Country: UK	None	Cross-sectional	*N* = 82	Generic preference-based	EQ-5D	Population mean	Only completed questionnaires used	NR
Patients with DTC who had undergone a total or near total thyroidectomy and RAI	Ratki [29]	Population: patients with DTC treated with RAI Country: Iran	None	Cross-sectional	*N* = 435	Patient-reported survey	EORTC QLQ-30	Whole group mean reported	94.5%	NR
Patients who were hospitalised for radioiodine administration and were hypothyroid as a result of short-term thyroid hormone withdrawal	Tagay [31] and Tagay [32]	Population: Patients with DTC hospitalised for RAI or whole body scanning and outpatients post-RAI Country: Germany	RAI	Cross-sectional	*N* = 136 (completing)	Patient-reported survey	SF-36	Whole group mean, summary scores for the two groups studied and the inpatient group split into those receiving RAI and those receiving a diagnostic body scan	96%	Discarded incomplete responses
Patients with DTC; most had undergone surgery and RAI	Tan [33]	Population: Patients with PTC or FTC Country: Singapore	None during study period	Prospective	*N* = 152	Patient-reported survey	SF-36	Whole group mean reported	52.4%	8 removed due to incomplete responses
Thyroid cancer survivors	Wang [52]	Population: majority had DTC Country: China	None	Population-based survey	*N* = 965	Patient-reported survey	SF-36 & EORTC QLQ-C30	SF-36: in 10-year age brackets	62%	According to SF-36 and EORTC QLQ-C30 developer’s guidelines
RAI and surgery										
Patients attending follow-up clinic for a whole body scan after initial surgery and RAI Levothyroxine withdrawal is necessary prior to the whole body scan	Botella-Carretero [21]	Population: thyroid cancer post-surgery during levothyroxine withdrawal Country: Spain	Post-thyroidectomy	Prospective	*N* = 18	Patient-reported survey	SF-36	Patient group at baseline and two follow-up points	Only respondents with completed questionnaires were included	NR
Patients with biopsy positive PTC	Economopoulos (abstract) [35]	Population: patients with PTC Country: US	Surgery for thyroid cancer	Prospective	*N* = 55	Generic preference-based	EQ-5D	Change from baseline to post-op and 6 month follow-up	60% (both pre-op and post-op questionnaires completed)	NR
HRQoL estimates elicited from physicians and nurses who care for people with thyroid cancer	Esnaola [36]	Populations: patients with PTC Country: USA	Thyroid lobectomy and total thyroidectomy	Decision analysis with TTO to elicit utility values	*N* = 15	TTO—interviews	Vignette health states evaluated via interview	Systemic recurrence of thyroid cancer and four disease-free states after surgical procedures	Only respondents with completed questionnaires were included	N/A
HRQoL data which were routinely collected in a nuclear medicine department of patients with DTC attending for RAI	Gamper [23]	Population: DTC prior to RAI treatment Country: Austria	RAI	Retrospective analysis of data on file	*N* = 241 (total patients)	Patient-reported survey	EORTC QLQ-30	Mean across baseline and all follow-up time points	72%	92 patients excluded with only one assessment
Patients undergoing surgery for papillary thyroid cancer	Gou [20]	Population: patients with PTC Country: China	Surgery for thyroid cancer	Prospective observational	*N* = 186	Patient-reported survey	SF-36	Mean across baseline and four points of follow-up	Only respondents with completed questionnaires were included	N/A
Patients undergoing thyroidectomy of two types	Huang [26]	Population: patients with PTC undergoing thyroidectomy Country: China	Endoscopic thyroidectomy (experimental group) Open thyroidectomy (control group)	Prospective	*N* = 198	Patient-reported survey	SF-36	Experimental and control groups at two time points post-surgery (1 month and 6 months)	Only respondents with completed questionnaires were included	N/A
Patients undergoing thyroidectomy	Kebebew [37]	Population: patients with low-risk DTC Country: USA	Thyroidectomy	Cross-sectional	*N* = 42	Standard gamble	No specific tool used	Thyroidectomy complications, DTC recurrence, and DTC mortality for low-risk patients	Only respondents with completed questionnaires were included	NR
Patients undergoing thyroidectomy of two types	Lee [27]	Population: patients with PTC undergoing thyroid lobectomy Country: Korea	Endoscopic thyroidectomy (experimental group) Open thyroidectomy (control group)	Prospective	*N* = 308	Patient-reported survey	EORTC QLQ-30	Experimental and control groups at baseline and three time points post-surgery (1 month and 6 months)	44%	NR
Patients undergoing surgery for papillary thyroid cancer	Lubitz [38]	Population: patients with PTC Country: US	Thyroidectomy	Prospective	*N* = 117	Generic preference-based	EQ-5D	Pre-treatment and then at two different follow-up times	48%	22 cases of missing data that were dealt with using a hot deck procedure stratified by sex, age group and survey time point
Patients with newly diagnosed PTC or FTC who had undergone thyroidectomy surgery in the previous 2 weeks	Pacini [28]	Population: patients treated with ablation for remnant thyroid; two groups one in hypothyroid one in euthyroid states Country: Europe and North America	Thyroid remnant ablation	Prospective	*N* = 63	Patient-reported survey	SF-36	Baseline at 4 week follow-up for both groups	Only respondents with completed questionnaires were included	N/A
Patients who had been treated for DTC with RAI, with or without surgery between 2009 and 2013 but were free of thyroid cancer at the time of the study	Rogers [19]	Population: patients with DTC who had been treated with RAI but were disease-free at the time of the study Country: UK	None	Prospective	*N* = 169	Patient-reported survey	EORTC QLQ-30	Whole group mean and results in age groups reported	68%	NR
Patients scheduled to undergo thyroid surgery	Shah [30]	Population: patients with well-DTC Country: Canada	Thyroid surgery	Prospective	*N* = 76	Patient-reported survey	SF-36	Pre-operatively and at 3 time points post-operatively	71% at 12 month follow-up	NR
Patients with DTC requiring radioiodine	SMC [45] Blamey [56] Haugen [57]	SMC (2015)b population: patients medullary with thyroid cancer Haugen et al. (1999) population is patients with DTC Country: USA	Recombinant human TSH	SMC cites Blamey et al. (2005), which is a literature review that references Haugen et al. (1999) as the source of utility data. The values are not found in the Haugen et al. (1999) publication	*N* = 229	SF-36	SF-36 converted to EQ-5D within the study with mapping	Progressed and non-progressed disease	NR	NR
Drug treatments										
Patients treated with thyroxine but disease-free at the time of analysis	Crevenna [22]	Population: patients with non-metastatic thyroid cancer being treated with thyroxine Country: Germany	Thyroxine	Cross-sectional	*N* = 150	Patient-reported survey	SF-36	Difference from utility values of general population without cancer	100%	Nurse present when patient filling out questionnaires to avoid missing answers
Patients with locally advanced or metastatic RR-DTC	Schlumberger (abstract) [48] SMC [50]	Population: RR-DTC Country: Multinational	Sorafenib	Phase III trial	*N* = 417 (SMC submission reports *N* = 416)	Generic preference-based	EQ-5D	Treatment effect	96%	NR
Patients with locally advanced or metastatic RR-DTC Did not directly collect utility data	Tremblay (abstract) [49]	Population: RR-DTC Country: multinational (details of countries not included in abstract)	Lenvatinib or sorafenib	Combined analysis of vignette utility values (Fordham et al. [46]) and the effectiveness evidence from clinical trials	NR	TTO vignettes	No specific tool used	Disutility from lenvatinib treatment, disutility from sorafenib treatment	NR	NR
Patients with DTC; cross-sectional element provided baseline HRQoL data for a total DTC group and a euthyroid group as a comparative control Prospective patients were randomised to the exercise training intervention or a control group	Vigario [34]	Population: patients with DTC on levothyroxine (subclinical hyperthyroidism) Country: Brazil	Supervised exercise training programme	Both cross-sectional and prospective elements	Subclinical hyperthyroidism (*N* = 33) Euthyroid subjects (*N* = 49)	Patient-reported survey	SF-36	Baseline HRQoL from the cross-sectional element of the study and 3 month follow-up for the prospective groups	100% at baseline and 3 month follow-up	NR
GTC										
Populations not assessed for a specific intervention										
Patients treated for thyroid cancer	Husson [39] Mols [9]	Population: patients with a diagnosis of thyroid cancer on a national registry Country: Holland	None	Cross-sectional	*N* = 307 (*N* = 293 in Mols)	Patient-reported survey	EORTC QLQ-30	HRQoL according to time since diagnosis, sub-groups according to patient age	86%	NR
Patients who had filled in a questionnaire measuring optimism and the SF-36 during their time being treated for thyroid cancer	Kung [40]	Population: patients with thyroid cancer. Most (65%) had disease stage 1 Country: USA	None	Retrospective	*N* = 104	Patient-reported survey	SF-36	The population is split into four groups according to their level of optimism	N/A Retrospective identification	NR
Patients who had been treated at hospital for thyroid cancer	Roberts [41]	Population: patients who had previously been treated for thyroid cancer Country: USA	None	Cross-sectional	*N* = 62	Patient-reported survey	EORTC QLQ-30	Whole group mean reported	43%	NR
Patients who answered ‘yes’ to a question about receiving a medical diagnosis of thyroid cancer	Ryu and Hwang [53]	Population: national survey data which included some thyroid cancer survivors Country: South Korea	None	Retrospective analysis of data on file	*N* = 125	Generic preference-based	EQ-5D	Whole group mean	Only respondents with completed questionnaires were included	N/A
Broad population of people with thyroid cancer	Singer [43]	Population: patients with thyroid cancer admitted to a rehabilitation clinic Country: Germany	None	Cross-sectional	*N* = 121	Patient-reported survey	EORTC QLQ-30	HRQoL according to type of thyroid cancer, by sex and according to a range of prognostic indicators	NR *N* = 121 is responders only	Missing values imputed
RAI and Surgery										
Patients enrolled on the ESTIMABL trial in two groups according to TSH and RAI method	Borget [44]	Population: patients undergoing TSH stimulating methods and RAI Country: multinational	RAI	Prospective	*N* = 752	Generic preference-based	EQ-5D	Mean utility values by method of stimulation and RAI at the time of RAI	91%	The number missing for each level is reported in a table in the publication
Drug treatments										
Patients undergoing a whole body scan as part of their thyroid cancer treatment	Schroeder [42]	Population: patients with thyroid cancer Country: USA	Whole body scanning and thyroxine	Prospective	*N* = 229	Patient-reported survey	SF-36	Baseline while on levothyroxine therapy, after recombinant human thyroid stimulant therapy while still on levothyroxine and on the day of the body scan or after thyroid hormone withdrawal but on the day of the body scan	98%	NR

*DTC* Differentiated thyroid cancer, *EORTC QLQ-30* European organisation for research and treatment of cancer 30-item quality-of-life questionnaire, *EQ-5D* EuroQol questionnaire 5-dimension, *FTC* follicular thyroid cancer, *N/A* not applicable, *NR* not reported, *PTC* papillary thyroid cancer, *RAI* radioiodine treatment, *RR-DTC* radioiodine-refractory differentiated thyroid cancer, *SF-36* 36-item Short-form health survey, *TSH* thyroid stimulating hormone, *UK* United Kingdom, *USA* United States of America

In 21 of the included studies (24 publications) [18–21, 24–28, 30, 33–36, 38, 42, 44, 46–48, 50–52, 54], utility values for thyroid cancer came from the prospective collection of HRQoL data; some of these data were collected as part of a clinical trial [48, 50].

Six studies (seven publications) [35, 38, 44, 48, 50, 51, 53] measured HRQoL using the EQ-5D-3L. In 23 studies, HRQoL was measured using either the SF-36 or the EORTC QLQ-30 and therefore mapping to the EQ-5D-3L was required (which comprised of 29 publications) [9, 18–34, 39–43, 45, 52, 54–57]. Mapping was conducted from the SF-36 in 16 studies and from the EORTC QLQ-30 in 7 studies. In one study [56], the mapping had been conducted by the study authors, therefore, we conducted mapping in 22 studies. In the remaining four studies (five publications), SG [37] and TTO [36, 46, 47, 49] techniques were used to estimate utility values. No studies using the HUI in this population were identified.

The reporting of the pain domain of the EORTC QLQ-30 was missing from some studies which meant these studies could not be included in the review; the mapping algorithm includes the pain domain and requires a complete set of domains to be used to estimate utility. Only three [19, 46, 47, 51] of the studies had an entirely UK-based population; this included only one [19] of the mapped studies. The regression equations are based on mapping to the EQ-5D-3L UK value set but, as not all of the HRQoL data were collected in the UK (27 studies with data collected completely outside of the UK), there are some potential inconsistencies in the results. If, for example, there is a tendency for quality-of-life estimates to be higher for a non-UK population, then any evidence used from these populations will generate higher utility estimates when mapped to the EQ-5D-3L UK value set.

Seven studies [39–44, 52] included patients with different sub-types of thyroid cancer; only one of these studies [43] provided utility estimates for each of the different sub-types.

Symptoms and comorbidities have a negative impact on HRQoL. Surgery has an initial impact on HRQoL that seems to resolve over time. A ceiling effect, where patients report full-health on the utility scale, with 51% of the population reporting full-health on the EQ-5D, was reported in one study [35]. The utility values are collated in Table 3.

**Table 3:** Included evidence utility values

Population	Publication	Health states	Utility values (SD)	Source	
DTC					
HRQoL assessed in a whole population (no specific intervention)					
RR-DTC	Fordham [46] and Kerr [47]	Stable/no response	0.80 (0.19)	Vignette values	
		Response to therapy (any form of treatment)	0.86 (0.15)		
		Progressive disease	0.50 (0.28)		
		Diarrhoea	0.42 (0.29)		
		Fatigue	0.72 (0.24)		
		Hand and foot syndrome	0.52 (0.30)		
		Alopecia	0.75 (0.21)		
DTC patients admitted to hospital	Haraj [55]	Whole group mean	0.494	SF-36 mapped to EQ-5D	
D TC (diagnosis 14–17 years previously)	Hedman [24]	At least one thyroid symptom	0.820	SF-36 mapped to EQ-5D	
		No thyroid symptoms	0.986		
DTC (diagnosis 14–17 years previously)	Hedman [25]	Total group	0.846	SF-36 mapped to EQ-5D	
		0 self-reported comorbidities	0.915		
		1 self-reported comorbidity	0.875		
		2 or more self-reported comorbidities	0.642		
		Self-reported recurrence	0.795		
		Concern of self-reported recurrence	0.815		
		No concern of self-reported recurrence	0.883		
		Negative view of life	0.658		
		Positive view of life	0.868		
DTC (diagnosis 14–17 years previously)	Hedman [18]	Major thyroid-related symptoms	0.711	SF-36 mapped to EQ-5D	
		Moderate thyroid-related symptoms	0.921		
		No thyroid-related symptoms	0.994		
DTC	Hedman [54]	At diagnosis		SF-36 mapped to EQ-5D	
		Negative view of life	0.710		
		Positive view of life	0.823		
		No fear of recurrence	0.857		
		Seldom fear recurrence	0.803		
		Often fear recurrence	0.757		
		Total	0.792		
		At one-year follow-up			
		Negative view of life	0.737		
		Positive view of life	0.870		
		No fear of recurrence	0.864		
		Seldom fear recurrence	0.863		
		Often fear recurrence	0.760		
		Presence of recurrence	0.745		
		Total	0.842		
		Moderate TSH suppression	0.855		
		Mild TSH suppression	0.745		
		No TSH suppression	0.878		
		Recurrence	0.745		
		No recurrence	0.853		
DTC with a diagnosis at least 6 months previously	McIntyre [51]	Whole DTC population	0.776 (0.26)	EQ-5D	
DTC patients post-thyroidectomy and RAI	Ratki [29]	Whole group mean	0.819	EORTC QLQ-30 mapped to EQ-5D	
DTC patients who are undergoing thyroid hormone withdrawal awaiting radioiodine treatment	Tagay [31]	Whole group mean	0.708	SF-36 mapped to EQ-5D	
DTC patients post-thyroidectomy and RAI	Tan [33]	Whole group mean	0.859	SF-36 mapped to EQ-5D	
Thyroid cancer survivors	Wang [52]	Whole population mean	0.927	EORTC QLQ-30 mapped to EQ-5D	
		Females		SF-36 mapped to EQ-5D	
		Aged 14–24	0.832		
		Aged 25–34	0.919		
		Aged 35–44	0.900		
		Aged 45–54	0.857		
		Aged 55–64	0.856		
		Aged ≥ 65	0.787		
		Males			
		Aged 25–34	0.927		
		Aged 35–44	0.921		
		Aged 45–54	0.920		
		Aged 55–64	0.921		
		Aged ≥ 65	0.861		
RAI and surgery					
DTC	Botella-Carretero [21]	Baseline (last day on current levothyroxine dose)	0.218	SF-36 mapped to EQ-5D	
		4–7 days after levothyroxine withdrawal	0.222		
		The day before administering RAI for whole body scanning	0.185		
	SMC [45] Blamey [56] Haugen [57]	Progressed disease	0.624	SF-36 mapped to EQ-5D values presented within the publications – no further mapping required	
		Non-progressed disease	0.796		
	Gamper [23]	Mean across pre-treatment with RAI and post-treatment	0.910	EORTC QLQ-30 mapped to EQ-5D-3L	
Patients with previous DTC	Rogers [19]	Whole group mean	0.916	EORTC QLQ-30 mapped to EQ-5D	
		Aged under 40	0.914		
		Aged between 40 and 49	0.911		
		Aged between 50 and 64	0.922		
		Aged over 65	0.900		
Patients with DTC undergoing thyroid surgery	Shah [30]	Baseline	0.856	SF-36 mapped to EQ-5D	
		3 months follow-up	0.824		
		6 months follow-up	0.899		
		12 months follow-up	0.947		
Patients with PTC	Economopoulos [35]	Change from pre-op to post-op	0.020 (0.15)	EQ-5D-3L	
		Change from pre-op to 6 month follow-up	− 0.016 (0.09)		
		Change from post-op to 6 month follow-up	− 0.023 (0.1)		
	Esnaola [36]	Disease-free after thyroid lobectomy	0.99	TTO	
		Disease-free after total thyroidectomy/radioiodine therapy	0.95		
		Disease-free after thyroid surgery/permanent complication	0.88		
		Disease-free after surgery for cervical recurrence	0.95		
		Systemic recurrence	0.60		
	Lubitz [38]	Pre-treatment	0.895 (0.103)	EQ-5D-5L	
		Change from baseline to post-op (2–4 weeks)	0.22 (0.12)		
		Change from post-op to follow-up (6–12 months post-surgery)	− 0.003 (0.11)		
PTC patients undergoing surgery	Gou [20]	Baseline	0.864	SF-36 mapped to EQ-5D-3L	
		1 month post-surgery	0.803		
		6 months post-surgery	0.870		
		12 months post-surgery	0.812		
		24 months post-surgery	0.825		
	Huang [26]	1 month post-surgery	0.791	SF-36 mapped to EQ-5D-3L	
		6 months post-surgery	0.972		
PTC patients undergoing open thyroidectomy (control group)		1 month post-surgery	0.676		
		6 months post-surgery	0.920		
	Kebebew [37]	Unilateral recurrent laryngeal nerve injury	0.627	Standard gamble	
		Bilateral recurrent laryngeal nerve injury	0.205		
		Hypoparathyroidism	0.778		
		DTC recurrence	0.54		
	Lee [27]	Pre-op	0.901	EORTC QLQ-30 mapped to EQ-5D	
		1 month post-surgery	0.887		
		3 months post-surgery	0.891		
		6 months post-surgery	0.901		
PTC undergoing endoscopic thyroidectomy (experimental group)		Pre-op	0.884		
		1 month post-surgery	0.852		
		3 months post-surgery	0.887		
		6 months post-surgery	0.866		
Euthyroid	Pacini [28]	Baseline	0.734	SF-36 mapped to EQ-5D	
		4 week follow-up	0.801		
Hypothyroid		Baseline	0.692		
		4 week follow-up	0.624		
Diagnostic patients	Tagay [32]	Group mean	0.676	SF-36 mapped to EQ-5D	
Therapy patients			0.718		
Inpatients			0.794		
Outpatients			0.705		
Drug treatments					
All thyroid cancer patients	Crevenna et al. [22]	Difference from the German general population	0.038	SF-36 mapped to EQ-5D	
		Diagnosed within one year	0.011		
RR-DTC	Schlumberger^a^ [48] SMC [50]	Sorafenib treatment effect	0.07	EQ-5D	
		Sorafenib arm of trial	0.72		
		BSC arm of trial	0.8		
	Tremblay [49]	Disutility from lenvatinib treatment	0.042	Vignette values	
		Disutility from sorafenib treatment	0.117		
Whole subclinical hyperthyroidism group (SCH)	Vigario [34]	Baseline	0.578	SF-36 mapped to EQ-5D	
Euthyroidism group		Baseline	0.833		
SCH intervention		Baseline	0.591		
		3 months post-intervention	0.800		
SCH control		Baseline	0.575		
		3 months post-intervention	0.608		
GTC					
Populations not assessed for a specific intervention					
Patients with thyroid cancer	Husson [39]	Less than 5 years since diagnosis	0.910	EORTC QLQ-30 mapped to EQ-5D	
		Between 5 and 10 years since diagnosis	0.902		
		More than 10 years since diagnosis	0.902		
	Kung [40]	Most optimistic	0.560	SF-36 mapped to EQ-5D	
		Second most optimistic	0.494		
		Third most optimistic	0.475		
		Most pessimistic	0.450		
	Roberts [41]	Whole group mean	0.941	EORTC QLQ-30 mapped to EQ-5D	
	Singer [43]	Papillary	0.835	EORTC QLQ-30 mapped to EQ-5D	
		Follicular	0.824		
		Medullary	0.875		
		Anaplastic	0.958		
		Tumour size 1	0.834		
		Tumour size 2	0.855		
		Tumour size 3	0.846		
		Tumour size 4	0.757		
		Lymph node metastases status 0	0.825		
		Lymph node metastases status 1	0.842		
		Distant metastases status 0	0.825		
		Distant metastases status 1	0.850		
		Men with thyroid cancer	0.878		
		Women with thyroid cancer	0.826		
		Thyroid cancer (whole group)	0.838		
Thyroid cancer survivors	Mols [9]	Adolescents and young adults	0.927	EORTC QLQ-30 mapped to EQ-5D	
		Middle-aged adults	0.897		
		Elderly	0.902		
	Ryu and Hwang [53]	Patients approximately 5 years from diagnosis	0.930	EQ-5D	
RAI and surgery					
Thyroid hormone withdrawal (THW)	Borget [44]	Mean at point of RAI administration	0.833 (0.192)	EQ-5D	
Recombinant human TSH (rhTSH)			0.849 (0.173)		
RAI 1.1 GBq			0.836 (0.184)		
RAI 3.7 GBq			0.846 (0.182)		
Drug treatments					
Patients with thyroid cancer	Schroeder [42]	Baseline; receiving levothyroxine therapy	0.542	SF-36 mapped to EQ-5D	
		After recombinant human thyroid stimulant therapy, still on levothyroxine and on the day of the body scan	0.544		
		After thyroid hormone withdrawal but on the day of the body scan	0.461		

*DTC* Differentiated thyroid cancer, *PTC* papillary thyroid cancer, *RAI* radioiodine treatment, *RR-DTC* radioiodine-refractory differentiated thyroid cancer, *SCH* subclinical hyperthyroidism, *SD* standard deviation

^a^DECISION trial quality-of-life analysis

## Discussion

The purpose of the review was to collate the utility values reported in the published literature and to highlight any populations for which utility estimates were not available. This review highlights the lack of utility values available for patients with ATC, MTC and the more severe form of DTC, radioiodine-refractory disease (RR-DTC). Evidence for patients with RR-DTC is limited to the data collected during the DECISION trial [58]. Evidence for ATC and MTC is also limited to one study [43].

Successful thyroidectomy surgery and radioiodine treatment can return patients to a HRQoL that approximates the average EQ-5D of the UK general population (0.856 mean for the UK population [59]) although, while receiving thyroid cancer treatment, HRQoL is negatively impacted [20, 21, 26, 27, 49].

The lowest estimate of utility for a health state (0.205) comes from patients with low-risk DTC [37] for bilateral recurrent laryngeal nerve injury as a complication of a thyroidectomy procedure. The utility estimates in this publication were generated using the SG method in a study of patients who had not all undergone the procedure nor experienced the complication.

Although ATC is the most severe sub-type of thyroid cancer, the only utility value for patients with ATC is higher than the values estimated for the other sub-types [43]. This utility estimate comes from a study [43] which compares HRQoL across the four most common thyroid cancer sub-types. Without alternative estimates for comparison, the impact of ATC on HRQoL remains uncertain.

The ‘ceiling effect’ of a large proportion of people valuing their health as the highest value that can be recorded on the EQ-5D-3L using the UK value set was observed in the study by Economopoulos [35] and may have also occurred in other studies but was not reported, as it is a common feature of HRQoL measurement with the EQ-5D-3L. Many patients with thyroid cancer do not experience significant symptoms and, after treatment, patients can be in long periods of remission [35]. This ‘ceiling effect’ may be problematic when trying to measure the impact of an intervention as if many patients are already recording the highest utility value (of one) then improvements to their HRQoL regardless of the intervention, cannot be measured. The use of the EQ-5D enables consistency across decisions that have been made in the past and therefore enables a level playing field for any new assessments of utility [11].

The Botella-Carretero [21] study produced results that seem anomalous when compared with the other utility values reported in this review, as they are much lower. This may be related to the withdrawal of the thyroid hormone; however, even the baseline HRQoL, measured before full hormone withdrawal, was substantially lower than any of the other utility estimates identified. Authors of the Schroeder et al. [42] study reported the poor HRQoL of patients being treated with levothyroxine or experiencing thyroid hormone withdrawal; this is in contrast to the studies by Borget et al. [44] and Tagay et al. [31] where a significant HRQoL decrement for these same health states was not demonstrated.

When conducting economic evaluations, judgements must be made about how well the available evidence represents the health states of interest [60]. These judgements are often based on two key elements: (a) does the health state from a published utility value encompass all aspects of the health state that is being considered in the economic evaluation (i.e. is the population in the published study sufficiently similar to the population being assessed) and (b) is the valuation methodology robust enough to produce a reliable estimate of that health state. The first element requires some input from topic specialists to allow the characteristics of the patients in each of the health states to be assessed, the second element relies on an evaluation of the methods used to derive utility values.

In addition to the review, we used mapping algorithms to convert the SF-36 and EORTC QLQ-30 questionnaires to the UK value set of the EQ-5D-3L. Using alternative algorithms would likely generate different results. However, we used algorithms considered to be the most appropriate for generating utility values for use in economic evaluations in the UK. The use of other mapping algorithms based on other HRQoL questionnaires would further broaden this catalogue. It should be noted that mapping algorithms have inherent flaws which make them less preferable for the estimation of utility values than direct measures of utility. In the absence of utility estimates for many of the health states experienced by patients with thyroid cancer, the mapped values presented in this study provide starting estimates.

The utility estimates contained within this review were not all derived from generic preference-based tools. However, when building an economic model or conducting an economic evaluation, it is necessary to attribute a utility value to each health state that is relevant to the population being studied. In the absence of an EQ-5D derived utility value for the population of interest, the use of a proxy value may be necessary. The applicability of the estimates available for each health state included in the evaluation needs to be assessed considering the population from which the estimate was obtained, the country of origin of the utility estimate, and the method used to obtain the estimate. Testing the importance of each of the parameters specified in an economic model using the results generated is important, and it may be preferable to use health state utility values which were obtained through less than perfect methods rather than omit health states completely due to a lack of values. Expert opinion can also be elicited to provide parameter estimates for use in any economic model or economic evaluation; however, the impact of proxy values on the overall results of the model should be evaluated using sensitivity analysis [61].

Authors of published studies with a similar aim to our review (i.e. collating utility values in a specific disease area [62–64]) have recommended a set of utility values to be used as a reference case for any economic models built in the same disease area. However, the lack of utility estimates for all sub-types, and the need to use proxy estimates for some health states mean identifying a set of standard values would make economic evaluations more consistent but less flexible in terms of the health states that could be included.

The populations described in the included studies are not all based in the UK, and therefore the appropriateness of mapping HRQoL responses to UK EQ-5D-3L value sets may be questioned. However, including only the studies estimating utility values in a British population would vastly minimise the scope of the catalogue as few health states would be available. Nevertheless, this review provides a catalogue of utility values for patients with thyroid cancer and at the same time offers details of the study methods used to elicit these values, allowing the end-user of the data to make an informed choice about which utility values to include in an economic evaluation. The information presented in the catalogue also enables health economists to quickly select individual publications of interest and choose to use alternative mapping algorithms, such as those to EQ-5D value sets other than the UK, if so desired.

Formal tools to assess the robustness of utility values estimated from a variety of study designs and HRQoL tools are currently not available, largely because the most appropriate estimate is dependent on the nature of the question posed. The most robust source of utility values is likely to be from prospective studies obtaining patient-reported outcomes using generic preference-based questionnaires [35, 38, 44, 48, 50]. However, there are several studies within this review that deviate from this ideal [1, 9, 18–34, 36, 37, 39–43, 45–47, 49, 51–57].

Studies that included only data describing a change in HRQoL from baseline were included. When combined with other baseline HRQoL estimates, these data could be useful for economic evaluations if the population from the original study and the modelled health state being considered were sufficiently similar. This study has some drawbacks. Some of the studies included had small sample sizes and some of the utility values presented are derived from mapping based on mean values. Utility values derived from HRQoL tools other than the EQ-5D-3L were mapped to the EQ-5D-3L UK value set in line with NICE’s reference case. However, it is plausible that disease specific HRQoL tools may provide a better insight into the utility experience by patients (e.g. for thyroid cancer patients the EORTC QLQ-C30 data could also be used to measure utility values based on the EORTC QLU-C10D). If available, the use of utilities derived from other HRQoL instruments may be more appropriate than expert opinion.

## Conclusions

This review provides published utility values for estimating the HRQoL of patients with thyroid cancer and includes in addition, utility estimates that could be estimated by mapping the SF-36 or the EORTC QLQ-30 scores to the EQ-5D-3L UK value set. There are few utility estimates for ATC and MTC sub-types specifically; however, utility values are available for DTC and for a broader mixed population of people with thyroid cancer, which include patients with different sub-types of thyroid cancer. Utility estimates are available for patients who have been treated with a wide range of thyroid interventions across different disease stages. The utility value estimates presented are, on the whole, consistent with each other and what would be expected from a clinical point of view, with the most severe sub-types having the lowest utility estimates, although based on only a few estimates, and the most invasive interventions having the biggest impact on utility. The available estimates in the catalogue provide a useful resource for health economists as they undertake economic evaluations of interventions for thyroid cancer.

## Electronic supplementary material

Below is the link to the electronic supplementary material.

Supplementary file1 (DOCX 19 kb)

Supplementary file2 (DOCX 19 kb)

## References

[1] Nguyen QT, Lee EJ, Huang MG, Park YI, Khullar A, Plodkowski RA (2015). Diagnosis and treatment of patients with thyroid cancer. American Health & Drug Benefits.

[2] La Vecchia C, Malvezzi M, Bosetti C, Garavello W, Bertuccio P, Levi F (2015). Thyroid cancer mortality and incidence: a global overview. International Journal of Cancer.

[3] Cancer Research UK Thyroid cancer incidence statistics. www.cancerresearchuk.org/health-professional/cancer-statistics/statistics-by-cancer-type/thyroid-cancer/incidence. Accessed 1st December 2019.

[4] Smittenaar CR, Petersen KA, Stewart K, Moitt N (2016). Cancer incidence and mortality projections in the UK until 2035. British Journal of Cancer.

[5] Busaidy NL, Cabanillas ME (2012). Differentiated thyroid cancer: Management of patients with radioiodine nonresponsive disease. Journal of Thyroid Research.

[6] Department of Health NHS conditions: thyroid cancer. https://www.nhs.uk/conditions/thyroid-cancer/. Accessed June 2020.

[7] Cancer Research UK Thyroid cancer treatment. https://www.cancerresearchuk.org/about-cancer/thyroid-cancer/treatment. Accessed 30th November 2019.

[8] Perros P, Boelaert K, Colley S, Evans C, Evans RM, Gerrard Ba G (2014). Guidelines for the management of thyroid cancer, third edition. Clinical Endocrinology.

[9] Mols F, Schoormans D, Smit JWA, Netea-Maier RT, Links TP, van der Graaf WTA (2018). Age-related differences in health-related quality of life among thyroid cancer survivors compared with a normative sample: results from the PROFILES Registry. Head & Neck.

[10] National Institute for Health and Care Excellence (NICE) Guide to the methods of technology appraisal 2013. Process and methods [PMG9]. Published date: April 2013. https://nice.org.uk/process/pmg9. Accessed 29 Jan 2020. 27905712

[11] Brazier J, Ara R, Azzabi I, Busschbach J, Chevrou-Séverac H, Crawford B (2019). Identification, review, and use of health state utilities in cost-effectiveness models: an ispor good practices for outcomes research task force report. Value in Health.

[12] Prescient and Strategic Intelligence (2018). Thyroid cancer therapeutics—pipeline analysis 2018, clinical trials & results, patent, designation, collaboration, and other developments. https://www.psmarketresearch.com/market-analysis/thyroid-cancer-therapeutics-pipeline-analysis Accessed 30 November 2018.

[13] CRD (2009). Systematic reviews: CRD's guidance for undertaking reviews in health care. https://www.york.ac.uk/media/crd/Systematic_Reviews.pdf

[14] Arber M, Garcia S, Veale T, Edwards M, Shaw A, Glanville JM (2017). Performance of ovid medline search filters to identify health state utility studies. International Journal of Technology Assessment in Health Care.

[15] Department of Health (2013). Guide to the methods of technology appraisal. In National Institute for Health and Care Excellence (Ed.). https://www.nice.org.uk/process/pmg9/resources/guide-to-the-methods-of-technology-appraisal-2013-pdf-2007975843781 27905712

[16] Ara R, Brazier J (2008). Deriving an algorithm to convert the eight mean SF-36 dimension scores into a mean EQ-5D preference-based score from published studies (where patient level data are not available). Value in Health.

[17] Crott R, Briggs A (2010). Mapping the QLQ-C30 quality of life cancer questionnaire to EQ-5D patient preferences. European Journal of Health Econonomics.

[18] Hedman C, Djarv T, Strang P, Lundgren CI (2017). Effect of thyroid-related symptoms on long-term quality of life in patients with differentiated thyroid carcinoma: a population-based study in Sweden. Thyroid.

[19] Rogers SN, Mepani V, Jackson S, Lowe D (2017). Health-related quality of life, fear of recurrence, and emotional distress in patients treated for thyroid cancer. British Journal of Oral and Maxillofacial Surgery.

[20] Gou J, Cheng W, Lei J, Pan Q, You W, Cai M (2017). Health-related quality-of-life assessment in surgical patients with papillary thyroid carcinoma: a single-center analysis from Mainland China. Medicine.

[21] Botella-Carretero JI, Galan JM, Caballero C, Sancho J, Escobar-Morreale HF (2003). Quality of life and psychometric functionality in patients with differentiated thyroid carcinoma. Endocrine-Related Cancer.

[22] Crevenna R, Zettinig G, Keilani M, Posch M, Schmidinger M, Pirich C (2003). Quality of life in patients with non-metastatic differentiated thyroid cancer under thyroxine supplementation therapy. Supportive Care in Cancer.

[23] Gamper EM, Wintner LM, Rodrigues M, Buxbaum S, Nilica B, Singer S (2015). Persistent quality of life impairments in differentiated thyroid cancer patients: results from a monitoring programme. European Journal of Nuclear Medicine & Molecular Imaging.

[24] Hedman C, Djarv T, Strang P, Lundgren CI (2015). Thyroid symptoms effect on general long-term quality of life among patients with differentiated thyroid carcinoma-a population based cohort study in Sweden. Thyroid.

[25] Hedman C, Djarv T, Strang P, Lundgren CI (2016). Determinants of long-term quality of life in patients with differentiated thyroid carcinoma—a population-based cohort study in Sweden. Acta Oncologica.

[26] Huang JK, Ma L, Song WH, Lu BY, Huang YB, Dong HM (2016). Quality of life and cosmetic result of single-port access endoscopic thyroidectomy via axillary approach in patients with papillary thyroid carcinoma. OncoTargets and Therapy.

[27] Lee MC, Park H, Lee BC, Lee GH, Choi IJ (2016). Comparison of quality of life between open and endoscopic thyroidectomy for papillary thyroid cancer. Head & Neck.

[28] Pacini F, Ladenson PW, Schlumberger M, Driedger A, Luster M, Kloos RT (2006). Radioiodine ablation of thyroid remnants after preparation with recombinant human thyrotropin in differentiated thyroid carcinoma: results of an international, randomized, controlled study. Journal of Clinical Endocrinology & Metabolism.

[29] Ratki SKR, Fallahi B, Saghari M, Ardekani AE, Beiki D, Mirabzade A (2012). Quality of life in patients with differentiated thyroid carcinoma treated by radioactive Iodine-131. Iranian Journal of Nuclear Medicine.

[30] Shah MD, Witterick IJ, Eski SJ, Pinto R, Freeman JL (2006). Quality of life in patients undergoing thyroid surgery. Journal of Otolaryngology.

[31] Tagay S, Herpertz S, Langkafel M, Erim Y, Bockisch A, Senf W (2006). Health-related quality of life, depression and anxiety in thyroid cancer patients. Quality of Life Research.

[32] Tagay S, Herpertz S, Langkafel M, Erim Y, Freudenberg L, Schopper N (2005). Health-related quality of life, anxiety and depression in thyroid cancer patients under short-term hypothyroidism and TSH-suppressive levothyroxine treatment. European Journal of Endocrinology.

[33] Tan LG, Nan L, Thumboo J, Sundram F, Tan LK (2007). Health-related quality of life in thyroid cancer survivors. Laryngoscope.

[34] Vigario Pdos S, Chachamovitz DS, Teixeira Pde F, Rocque Mde L, Santos ML, Vaisman M (2014). Exercise is associated with better quality of life in patients on TSH-suppressive therapy with levothyroxine for differentiated thyroid carcinoma. Arquivos Brasileiros de Endocrinologia e Metabologia.

[35] Economopoulos KP, Benitez L, Gazelle G, Halpern EF, Donelan K, Swan J (2014). Responsiveness of generic utilities indices for patients undergoing surgery for thyroid cancer. Thyroid.

[36] Esnaola NF, Cantor SB, Sherman SI, Lee JE, Evans DB (2001). Optimal treatment strategy in patients with papillary thyroid cancer: a decision analysis. Surgery.

[37] Kebebew E, Duh QY, Clark OH (2000). Total thyroidectomy or thyroid lobectomy in patients with low-risk differentiated thyroid cancer: surgical decision analysis of a controversy using a mathematical model. World Journal of Surgery.

[38] Lubitz CC, De Gregorio L, Fingeret AL, Economopoulos KP, Termezawi D, Hassan M (2017). Measurement and variation in estimation of quality of life effects of patients undergoing treatment for papillary thyroid carcinoma. Thyroid.

[39] Husson O, Haak HR, Buffart LM, Nieuwlaat WA, Oranje WA, Mols F (2013). Health-related quality of life and disease specific symptoms in long-term thyroid cancer survivors: a study from the population-based PROFILES registry. Acta Oncologica.

[40] Kung S, Rummans TA, Colligan RC, Clark MM, Sloan JA, Novotny PJ (2006). Association of optimism-pessimism with quality of life in patients with head and neck and thyroid cancers. Mayo Clinic Proceedings.

[41] Roberts KJ, Lepore SJ, Urken ML (2008). Quality of life after thyroid cancer: an assessment of patient needs and preferences for information and support. Journal of Cancer Education.

[42] Schroeder PR, Haugen BR, Pacini F, Reiners C, Schlumberger M, Sherman SI (2006). A comparison of short-term changes in health-related quality of life in thyroid carcinoma patients undergoing diagnostic evaluation with recombinant human thyrotropin compared with thyroid hormone withdrawal. Journal of Clinical Endocrinology & Metabolism.

[43] Singer S, Lincke T, Gamper E, Bhaskaran K, Schreiber S, Hinz A (2012). Quality of life in patients with thyroid cancer compared with the general population. Thyroid.

[44] Borget I, Bonastre J, Catargi B, Deandreis D, Zerdoud S, Rusu D (2015). Quality of life and cost-effectiveness assessment of radioiodine ablation strategies in patients with thyroid cancer: results from the randomized phase III ESTIMABL trial. Journal of Clinical Oncology.

[45] NHS Scotland (2015). Cabozantinib SMC No. (1022/15). In Scottish Medicines Consortium (Ed.).

[46] Fordham BA, Kerr C, de Freitas HM, Lloyd AJ, Johnston K, Pelletier CL (2015). Health state utility valuation in radioactive iodine-refractory differentiated thyroid cancer. Patient Preference & Adherence.

[47] Kerr C, Fordham B, de Freitas HM, Pelletier CL, Lloyd A (2014). Health state utility valuation in radio-iodine refractory differentiated thyroid cancer (RR-DTC). Value in Health.

[48] Schlumberger M, Jarzab B, Elisei R, Siena S, Bastholt L, De La Fouchardiere C (2013). Phase III randomized, double-blinded, placebocontrolled trial of sorafenib in locally advanced or metastatic patients with radioactive iodine (RAI)-refractory differentiated thyroid cancer (DTC)-exploratory analyses of patient-reported outcomes. Thyroid.

[49] Tremblay G, Lloyd A, Majethia U, Pelletier C, Forsythe A, Briggs A (2015). Incremental quality adjusted life years (QALY) analysis in absence of head to head and health related quality of life (hrqol) data: a case study in thyroid cancer. Value in Health.

[50] NHS Scotland (2015). Sorafenib SMC No.(1055/15). In Scottish Medicines Consortium (Ed.).

[51] McIntyre C, Jacques T, Palazzo F, Farnell K, Tolley N (2018). Quality of life in differentiated thyroid cancer. International Journal of Surgery.

[52] Wang T, Jiang M, Ren Y, Liu Q, Zhao G, Cao C (2018). Health-related quality of life of community thyroid cancer survivors in Hangzhou. China. Thyroid.

[53] Ryu M, Hwang JI (2019). Cancer site differences in the health-related quality of life of Korean cancer survivors: results from a Population-based Survey. Public Health Nursing.

[54] Hedman C, Djarv T, Strang P, Lundgren CI (2018). Fear of recurrence and view of life affect health-related quality of life in patients with differentiated thyroid carcinoma: a Prospective Swedish Population-Based Study. Thyroid.

[55] Haraj NE, Bouri H, El Aziz S, Nani S, Habti N, Chadli A (2019). Evaluation of the quality of life in patients followed for differentiated cancer of the thyroid. Annales d Endocrinologie.

[56] Blamey S, Barraclough B, Delbridge L, Mernagh P, Standfield L, Weston A (2005). Using recombinant human thyroid-stimulating hormone for the diagnosis of recurrent thyroid cancer. Australia and New Zealand Journal of Surgery.

[57] Haugen B, Pacini F, Reiners C, Schlumberger M, Ladenson P, Sherman S, Cooper D, Graham K, Braverman L, Skarulis M, Davies T, Degroot L, Mazzaferri E, Daniels G, Ross D, Luster M, Samuels M, Becker D, Maxon HI, Cavalieri R, Spencer C, Mcellin K, Weintraub B, Ridgway E (1999). A comparison of recombinant human thyrotropin and thyroid hormone withdrawal for the detection of thyroid remnant or cancer. The Journal of Clinical Endocrinology & Metabolism.

[58] Brose MS, Nutting CM, Jarzab B, Elisei R, Siena S, Bastholt L (2014). Sorafenib in radioactive iodine-refractory, locally advanced or metastatic differentiated thyroid cancer: a randomised, double-blind, phase 3 trial. Lancet.

[59] Janssen B, Szende A, Szende A, Janssen B, Cabases J (2014). Population Norms for the EQ-5D. Self-reported population health: an international perspective based on EQ-5D.

[60] Ara R, Brazier J, Peasgood T, Paisley S (2017). The identification, review and synthesis of health state utility values from the literature. Pharmacoeconomics.

[61] Weinstein MC, O'Brien B, Hornberger J, Jackson J, Johannesson M, McCabe C (2003). Principles of good practice for decision analytic modeling in health-care evaluation: report of the ISPOR Task Force on Good Research Practices-Modeling Studies. Value in Health.

[62] Brazier, J., Green, C., & A Kanis, J. (2002). A systematic review of health state utility values for osteoporosis-related conditions (Vol. 13). 10.1007/s001980200107 12378365

[63] Tonmukayakul U, Le LK-D, Mudiyanselage SB, Engel L, Bucholc J, Mulhern B (2018). A systematic review of utility values in children with cerebral palsy. Quality of Life Research.

[64] Forsythe A, Brandt PS, Dolph M, Patel S, Rabe A, Tremblay G (2018). Systematic review of health state utility values for acute myeloid leukemia. ClinicoEconomics and Outcomes Research.

