# Supplementary material for: A systematic review of health state utility values for thyroid cancer
Source: Qual Life Res. 2020 Oct 24;30(3):675–702. doi: 10.1007/s11136-020-02676-2 (PMC7952343; doi:10.1007/s11136-020-02676-2)
Supplement: Supplementary file 1 — Supplementary file1 (DOCX 19 kb) [file 11136_2020_2676_MOESM1_ESM.docx]

# Appendix A

**Search strategy (MEDLINE)**

| 1. exp Thyroid Neoplasms/ |  |
| --- | --- |
| 2. ((thyroid* or papillar* or follicular*) adj4 (Neoplasm* or Cancer* or Carcinoma* or Adenocarcinom* or Tumour* or Tumor* or Malignan* or Lump* or adenoma*)).tw. |  |
| 3. (DTC or FTC or PTC).tw. |  |
| 4. adenocarcinoma, follicular/ or carcinoma, papillary, follicular/ or adenocarcinoma, papillary/ |  |
| 5. 1 or 2 or 3 or 4 |  |
| 6. Quality-Adjusted Life Years/ |  |
| 7. Value of Life/ |  |
| 8. (qaly$ or qald$ or qale$ or qtime$).ti,ab,kf. |  |
| 9. (quality adjusted or adjusted life year$).ti,ab,kf. |  |
| 10. disability adjusted life.ti,ab,kf. |  |
| 11. daly$1.ti,ab,kf. |  |
| 12. ((index adj3 wellbeing) or (quality adj3 wellbeing) or qwb).ti,ab,kf. |  |
| 13. (multiattribute$ or multi attribute$).ti,ab,kf. |  |
| 14. utility.ab. /freq=2 |  |
| 15. utilities.ti,ab,kf. |  |
| 16. disutili$.ti,ab,kf. |  |
| 17. (HSUV or HSUVs).ti,ab,kf. |  |
| 18. health$1 year$1 equivalent$1.ti,ab,kf. |  |
| 19. (hye or hyes).ti,ab,kf. |  |
| 20. (hui or hui1 or hui2 or hui3).ti,ab,kf. |  |
| 21. (illness state$1 or health state$1).ti,ab,kf. |  |
| 22. (eq-sdq or eqsdq).ti,ab,kf. |  |
| 23. (short form$ or shortform$).ti,ab,kf. |  |
| 24. (sf36$ or sf 36$ or sf thirtysix or sf thirty six).ti,ab,kf. |  |
| 25. (sf12 or sf 12 or sf twelve or sftwelve).ti,ab,kf. |  |
| 26. (sf16 or sf 16 or sf sixteen or sfsixteen).ti,ab,kf. |  |
| 27. (sf20 or sf 20 or sf twenty or sftwenty).ti,ab,kf. |  |
| 28. (15D or 15-D or 15 dimension).ti,ab,kf. |  |
| 29. (standard gamble$ or sg).ti,ab,kf. |  |
| 30. (time trade off$1 or time tradeoff$1 or tto or timetradeoff$1).ti,ab,kf. |  |
| 31. (utility adj3 (score$1 or scoring or valu$ or measur$ or evaluat$ or scale$1 or instrument$1 or weight or weights or weighting or information or data or unit or units or health$ or life or estimat$ or elicit$ or disease$ or mean or cost$ or expenditure$1 or gain or gains or loss or losses or lost or analysis or index$ or indices or overall or reported or calculat$ or range$ or increment$ or state or states or status)).ti,ab,kf. |  |
| 32. (euro qual or euro qual5d or euro qol5d or eq-5d or eq5-d or eq5d or euroqual or euroqol or euroqual5d or euroqol5d).ti,ab,kf. |  |
| 33. (sf6 or sf 6 or sf6d or sf 6d or sf six or sfsix or sf8 or sf 8 or sf eight or sfeight).ti,ab,kf. |  |
| 34. "Quality of Life"/ |  |
| 35. (qualit* adj2 life).tw. |  |
| 36. or/6-35 |  |
| 37. 5 and 36 |  |
