# Supplementary material for: A systematic review of health state utility values for thyroid cancer
Source: Qual Life Res. 2020 Oct 24;30(3):675–702. doi: 10.1007/s11136-020-02676-2 (PMC7952343; doi:10.1007/s11136-020-02676-2)
Supplement: Supplementary file 2 — Supplementary file2 (DOCX 19 kb) [file 11136_2020_2676_MOESM2_ESM.docx]

# Appendix B

Domains and response levels from health-related quality of life questionnaires included

| **EQ-5D-3L** | **EQ-5D-5L** | **EORTC QLQ-30** | **SF-36** |
| --- | --- | --- | --- |
| **Domains** | | | |
| Mobility | Mobility | Five function domains (physical, role, emotional, cognitive, and social) | Physical functioning (10 items) |
| Self-care | Self-care | Three symptom scales (fatigue, nausea or vomiting, and pain) | Physical role limitations (4 items) |
| Usual activities | Usual activities | Six single (mainly symptoms) items (dyspnoea, insomnia, appetite loss, constipation, diarrhoea, and financial difficulties) | Bodily pain (2 items) |
| Pain/ discomfort | Pain/ discomfort |  | General health perceptions (5 items) |
| Depression/ anxiety | Depression/ anxiety |  | Energy/vitality (4 items) |
|  |  | Global health status/ quality of life scale (two items) | Social functioning (2 items) |
|  |  |  | Emotional role limitations (3 items) |
|  |  |  | Mental health (5 items) |
| **Response level** | | | |
| 3 (no problems, some problems, extreme problems) | 5 (no problems, slight problems, moderate problems, severe problems, unable to/ extreme problems) | 4 levels for 28 items (not at all, a little, quite a bit, very much); 7 levels for 2 items (ranging from 1=very poor to 7=excellent) | Response level varies for the different items ranging from 2 to 6 options |

EORTC QLQ-30=European Organisation for Research and Treatment of Cancer 30-item quality of life questionnaire; EQ-5D=EuroQol questionnaire 5-dimension; SF-36=36-item Short-form health survey
